# Mung bean proteins and peptides: nutritional, functional and bioactive properties

**DOI:** 10.29219/fnr.v62.1290

**Published:** 2018-02-15

**Authors:** Zhu Yi-Shen, Sun Shuai, Richard FitzGerald

**Affiliations:** 1 College of Biotechnology and Pharmaceutical Engineering, Nanjing Tech University, Nanjing, China; 2 Life Science Department, University of Limerick, Limerick, Ireland

**Keywords:** nutrition, protein extraction, functionality, globulins, angiotensin converting enzyme inhibitory activity, trypsin inhibitory activity, anti-fungal activity

## Abstract

To date, no extensive literature review exists regarding potential uses of mung bean proteins and peptides. As mung bean has long been widely used as a food source, early studies evaluated mung bean nutritional value against the Food and Agriculture Organization of the United Nations (FAO)/the World Health Organization (WHO) amino acids dietary recommendations. The comparison demonstrated mung bean to be a good protein source, except for deficiencies in sulphur-containing amino acids, methionine and cysteine. Methionine and cysteine residues have been introduced into the 8S globulin through protein engineering technology. Subsequently, purified mung bean proteins and peptides have facilitated the study of their structural and functional properties. Two main types of extraction methods have been reported for isolation of proteins and peptides from mung bean flours, permitting sequencing of major proteins present in mung bean, including albumins and globulins (notably 8S globulin). However, the sequence for albumin deposited in the UniProt database differs from other sequences reported in the literature. Meanwhile, a limited number of reports have revealed other useful bioactivities for proteins and hydrolysed peptides, including angiotensin-converting enzyme inhibitory activity, anti-fungal activity and trypsin inhibitory activity. Consequently, several mung bean hydrolysed peptides have served as effective food additives to prevent proteolysis during storage. Ultimately, further research will reveal other nutritional, functional and bioactive properties of mung bean for uses in diverse applications.

Many health organisations worldwide have recommended increased intake of plant-based foods to improve the prevention of chronic diseases and to improve overall human health. As a result, a variety of plant-based functional foods have been introduced into health care programmes (1). One such crop that has exhibited health benefits is mung bean [*Vigna radiata* (L.)], which is a summer pulse crop with a short growth cycle (70–90 days). It is a widely cultivated plant in many Asian countries as well as in dry regions of southern Europe and warmer parts of Canada and the United States. As an important plant-derived food resource (1), mung bean (2) is well known for its detoxification bioactivities. In addition, it has been used for treating numerous other conditions ranging from enhancement of human mental function to alleviation of heat stroke (3). The overall nutritional properties of mung beans have been recently reviewed by Dahiya et al. (4). Due to its high nutritional value, (5) especially in seeds, mung bean has served as an important food/feed source for humans and animals. Mung bean seeds contain about 20.97–31.32% protein (6), compared to 18–22% (7) and 20–30% (8) for the protein content in soy and kidney beans, respectively. Moreover, protein content of mung bean seeds is about twofold higher than in the cereal seed maize, with a lower storage protein content (7 to 10%) (9) and significantly higher protein content than observed for conventional root crops (10).

Although high levels of proteins and amino acids in mung beans (11) are believed to be the main contributors to its nutritional content, a low methionine content and the presence of trypsin inhibitor (12) in mung bean seed are thought to be responsible for its low protein efficiency ratio (PER). Meanwhile, mung bean proteins and peptides have also been reported to possess angiotensin-converting enzyme (ACE) inhibitory activity, as well as anti-fungal and/or antibacterial activities (3). Although major past use of mung bean seeds has been as a food resource, more recently mung bean extracts, especially protein and peptide isolates, have gained increasing attention for additional diverse applications.

## Nutritional properties of the mung bean proteins

As mentioned above, mung bean seeds are particularly rich in protein, containing about 20.97–31.32% protein content (6). Mubarak (13) reported a chemical score of 76% for mung bean amino acids, which was calculated based on the Food and Agriculture Organisation of the United Nations (FAO)/the World Health Organisation (WHO) (14) guidelines. Therefore, due to its high protein content and digestibility, consumption of mung bean seeds in combination with cereals has been recommended to significantly increase the quality of protein intake as part of a vegetarian diet (3). To characterise this nutritional content more specifically, Kudre et al. (10) analysed the protein composition of isolates from mung bean seeds. The total protein content in mung bean protein isolates (MBPI) was 87.8%, with a total amino acid content of 800.2 mg g^−1^ (Table 1). Essential amino acids constituted 43.5% of total amino acids in MBPI, whereas sulphur-containing amino acids constituted approximately 1.6% of total MBPI amino acids.

**Table 1:** Amino acids in mung bean protein isolates with levels comparing the ones adapted from FAO/WHO (15) guidelines: (10)]

MBPI levels	Amino acids	MBPI (mg g^−1^)	FAO/WHO (mg g^−1^)
Overview	Total amino acids	800.2	
	Total essential amino acids	348.2 (43.51%)^a^	
	Total aromatic amino acids	96.7 (12.08%)^a^	
	Total sulfur amino acids	13.0 (1.62%)^a^	
Higher levels	Phenylalanine + Tyrosine	90.3	63
	Leucine	74	66
	Lysine	62.4	58
	Valine	46.3	35
	Isoleucine	39.1	28
	Histidine	27.9	19
Lower levels	Threonine	28.4	34
	Methionine + cysteine	13	25
	Tryptophan	6.4	11
Not mentioned by the FAO/WHO	Glutamic acid/glutamine	125.4	
	Aspartic acid/asparagine	85.3	
	Arginine	64.4	
	Serine	38.5	
	Alanine	36.6	
	Glycine	32.2	
	Proline	30	

MBPI, mung bean protein isolates.

a Percent of amino acids, relative to total amino acids in MBPI.

Specifically, the essential amino acids such as leucine, lysine and phenylalanine/tyrosine were predominant, followed by valine, isoleucine and histidine (Table 1). In addition, the aromatic amino acid content of MBPI was 12.1%, in which phenylalanine and tyrosine constitutes 11.3% (90.3 of 800.2 mg g^−1^). Indeed, the total essential amino acid content of MBPI exceeds the FAO/WHO recommendations (15). Conversely, values for threonine, tryptophan and total sulphur-containing amino acids (methionine and cysteine) were nutritionally inadequate (Table 1).

The protein content of mung bean has been reported to be negatively correlated with the content of lysine and threonine (4), whereas the latter has been positively correlated with methionine content. These results suggest that increase in methionine content is accompanied by decreased total protein content in mung bean. Therefore, the reverse scenario of high protein content in mung bean seed probably reflects low methionine content (16, 17). In addition, low levels of threonine, tryptophan and sulphur-containing amino acids (methionine and cysteine), compared to the FAO/WHO recommended values (Table 1), were reported in MBPI. However, Khalil (18) reported that the threonine content was 140.88% of the value provided by the FAO/WHO, as compared to 83.53% reported by Kudre et al. (10).

Although mung bean seeds are rich in protein, the deficiency in the sulphur-containing amino acids (methionine and cysteine) places the nutritional quality of mung bean seeds on par with other legumes (19). To address the lack of sulphur-containing amino acids, methionine was successfully introduced into 8Sα globulin, a major mung bean protein, using protein engineering (20). Consequently, the nutritional quality of the modified protein containing increased methionine in terms of amino acid score improved from 41 to 145%. In a similar vein, Torio et al. (11) reported another protein engineering method that introduced free sulfhydryl groups and disulphide bonds to generate cysteine-modified mung bean 8Sα globulin to improve nutritional quality.

Meanwhile, the presence of hydrophobic amino acids has been reported to contribute greatly to the thermal and/or conformational stability of globulins to boost yield (10). Consequently, hydrophobic amino acid content increased to 53.1% in MBPI after substitution of charged amino acids with hydrophobic amino acids.

In addition to the amino acids evaluated using FAO/WHO (15) guidelines mentioned above, other important amino acids omitted from the guidelines should also be mentioned (Table 1). Three of these, glutamic acid, glutamine and arginine, are abundant in mung bean seeds and are thought to be important for brain development, exhibiting neuro-protective functions in infants (21). In addition, dietary glutamine can improve gastrointestinal barrier integrity by reducing systemic infections, and by stimulating lymphocyte proliferation, monocyte function and T cell 1 helper cytokine responses that may improve brain growth. Glutamine can also reduce systemic inflammation by decreasing the production of pro-inflammatory cytokines (IL-8 and IL-6). Arginine also has some benefits and has been shown to increase cerebral blood flow and increase nitric oxide production to help decrease necrotising enterocolitis incidence.

As mentioned above, non-genetically engineered mung bean proteins contain adequate amounts of most essential amino acids, with the exception of the sulphur-containing amino acids (methionine and cysteine). Methionine and cysteine have previously been obtained dietarily by sulphur-containing amino acids but deficient in lysine (4). A 7:3 ratio of rice protein has been recommended as an optimal ratio for consumption (22).

## Mung bean seed proteins

Mung bean seeds are rich in storage proteins, which account for about 85% of total protein (9). The crop’s major seed storage proteins include albumins, globulins and prolamins, which are soluble in water, in dilute saline and in alcohol–water mixtures, respectively (23). Globulin and albumin, which make up over 60 and 25% of total mung bean protein, respectively, represent the main mung bean storage proteins. Prolamin, however, has not yet been isolated and characterised from mung bean. Aside from these major proteins, components comprising the other 15% of mung bean protein components have not yet been extensively studied to date, except for trypsin inhibitor (24), non-specific lipid transfer proteins (nsLTP) (25) and thiamine-binding proteins (26) (Table 2).

**Table 2:** The composition of major protein fractions and individual protein components in mung bean [*Vigna radiata* (L.)]

Types	Proteins	Subunits	Molecular weight
Storage proteins [85% (9) in overall proteins]	Globulins [60% (9) in overall proteins]	Globulin 7S	28kD (27)
			16kD (27)
			8S_α_ 5.2kD (19)
		Globulin 8S	8S_α_’5.2kD (19)
			8S_β_ 5.2kD (19)
		Globulin 11S	40kD (28)
			24kD (27)
	Albumin [25% (9) in overall proteins]		
Other proteins (15% in overall proteins)	Trypsin inhibitor		14kD (24)
	Non-specific lipid transfer peptide (nsLTP)		9.03kD (25)
	Thiamine-binding proteins (TBP)		72.6kD (26)
	Others		

Three types of globulins present in mung bean seed have been characterised and are designated as basic-type (7S), vicilin-type (8S) and legumin-type (11S) globulins (5, 27), comprising 3.4%, 89.0% (5, 27) and 7.6% (w/w) of total mung bean globulin content, respectively (27).

In mature seeds, the major storage protein is 8S globulin, which is the most reported globulin in mung bean proteins (28). No disulphide linkages exist in 8S globulin, due to the lack of cysteine content (19, 27, 29). The 8S globulin consists of four subunits with molecular masses ranging from 26 to 60 kDa, as observed using SDS-PAGE analysis (16, 27, 29). Mendoza et al. (27) has determined the N-terminal amino acid sequences of the four mung bean 8S globulin subunits, which are EDKEEQ (60 kDa), IDAAEVSVSRGKNNPFYFNN (48 kDa), SKTLSSQNEPFNLRLN (32 kDa) and IDGAEVSVSRGKNNP (26 kDa).

Three highly conserved isoforms of the 8S globulin have been classified as 8Sα (UniProt ID: B1NPN8_VIGRA), 8Sα’ (UniProt ID: Q198W4_VIGRA) and 8Sβ (UniProt ID: Q198W3_VIGRA) (9, 19). Bernardo et al. (19) studied the amino acid sequence homologies of these three isoforms, which were found to be 91–92% between 8Sα and 8Sα’, 87% between 8Sα and 8Sβ, and 86–88% between 8Sα’ and 8Sβ. Another study showed, using the SignalP website server (30), that 8Sα, 8Sα’ and 8Sβ signal peptide sequences mapped to residues 1–25, 1–24 or 1–25, and 1–23, respectively.

The detailed structure of 8Sα globulin has been reported to consist of three subunits, each subunit containing two modules. X-ray crystallographic analysis has demonstrated that each module consists of a β-barrel domain and an extended loop domain (5). The overall 8Sα globulin structure exhibits 68% sequence identity and structural similarity (a root-mean-square deviation of 0.6 Å) with soybean β–conglycinin β (soybean 7S globulin), and both proteins share surface hydrophobicity characteristics. However, analysis of cavity size and other structural features derived from the mung bean 8Sα globulin crystal structure suggests that the thermal stability of 8Sα globulin is lower than that of soybean β–conglycinin β.

Methionine and cysteine residues have been introduced into the 8Sα globulin through protein engineering. Analysis of the methionine-modified mung bean protein, which can possess up to 10 additional methionine residues (20), indicates that the modified 8Sα globulin exhibits improved structural stability versus the wild-type protein, as assessed by differential scanning calorimetry (DSC). Notably, no allergenic potential has been identified in either wild-type or modified protein. Meanwhile, a report by Torio et al. (11) demonstrated improved structural stability and improved heat-induced gelation properties for cysteine-modified mung bean 8Sα globulin versus wild-type protein.

To our knowledge, only a limited number of reports covering the characteristics of mung bean 7S and 11S globulins exist. Mendoza et al. (27) used SDS-PAGE to show that the two bands yielded from 11S globulin were of 40 kDa and 24 kDa in size, whereas the two bands yielded from 7S globulin were of 28 kDa and 16 kDa in size. The N-terminal amino acid sequences for these four bands were determined to be NYVMNPAYVLMKPTQKDAAL (for the 28 kDa subunit) and STTVGHSGGTMIST (for the 16 kDa subunit) of 7S globulin and SSSSTNNRF [for the 40 kDa acidic subunit (29)] and GLEETIXSSK [for the 24 kDa basic subunit (29)] for the 11S globulin. The presence of disulphide bridges in these 7S and 11S globulins was demonstrated using SDS-PAGE with and without β-mercaptoethanol. The 7S and 11S globulins each exhibited only one band in the absence of β-mercaptoethanol, but two bands in the presence of the reducing agent. Meanwhile, mung bean globulins possessing higher 11S to 7S globulin ratios were reported to exhibit improved functionalities, for example, solubility and emulsifying activities (29).

Research on mung bean storage proteins has also provided information about this major group of globulins. In one study, Ericson et al. (16) used sucrose gradient centrifugation to show that 8S and 11S globulins account for approximately 85 and 15% of total globulins, respectively. In addition, the acidic nature of 11S globulins was shown to be less pronounced than that of 8S globulins, due to the greater relative prevalence of disulphide bridges between individual acidic and basic polypeptides in 11S versus 8S globulins (29).

Only a limited number of studies have been carried out regarding mung bean albumin, resulting in deposition of only one albumin sequence in the UniProt database (mung bean seed albumin, UniProt ID: Q43680_VIGRR). However, the reference cited for that entry was not accepted by *Plant Molecular Biology*. Another mung bean albumin sequence in the UniProt database (albumin 1, UniProt ID: ALB1_VIGRR) does not match with the mung bean sequence reported by Yamazaki et al. (31)*,* the reference cited in the UniProt sequence database.

## Functional properties of MBPI

Functional properties of proteins play a significant role as additives for food processing applications. Therefore, it is necessary to study the physicochemical characteristics of MBPI as food ingredients (32). Recently, several reports have been published regarding functional properties of mung bean proteins (33–35), including protein solubility, water absorption capacity (WAC), oil absorption capacity (OAC), foaming capacity (FC) and foam stability (FS), emulsifying activity (EA) and emulsifying stability (ES), and thermal properties. Consequently, such protein or peptide properties can improve the functionality of food processing applications; for example, the emulsifying property of the protein helps to stabilise emulsions, beverages or foams to prolong food shelf life (33). Functional improvements by MBPI would make it more applicable as food supplements.

### 1. Protein solubility

Solubility is considered as an important functional property of proteins, because it acts as a vital factor of the sensory quality attributes of foods (36). This property is the thermodynamic index of the equilibrium between protein–protein and protein–solvent interactions. Variations in factors, such as temperature, pH, ion strength, freezing, heating and drying, lead to changes in proteins’ structural conformations, which in turn affect protein functionality (37). Additionally, pH value is an important index of protein solubility to determine the behaviour of protein isolate in food process.

Due to electrostatic repulsion and hydration, the solubility of MBPI had been higher at pH values of 2, 10 and 12 than that of other pH values; for example, at pH 4, the lowest solubility was observed and aggregation occurs (38). Du et al. (35) determined that the minimum solubility of MBPI appeared at pH 4.6, which is the isoelectric point. The protein solubility of untreated MBPI was improved by additional heating from 61.5 to 65.6%, which is attributed to increases in charged residues on the MBPI surface, as a result of protein denaturation and/or unfolding (32). Butt and Batool (39) reported that protein solubility of MBPI was 72% at pH 7. Kudre et al. (10) determined maximum protein solubility of MBPI as 70.6% at pH 10 (Table 3). They also demonstrated that solubility of MBPI could be improved with the aid of NaCl at an appropriate concentration. Liu et al. (33) demonstrated that the solubility of mung bean 8S globulin ranged from 51.59 to 74.33%, with an average of 64.21%.

**Table 3:** Functional properties of mung bean protein isolates

	Protein solubility (%)	Water absorption capacity	Oil absorption capacity	Emulsion activity (%)	Emulsion stability (%)	Foam capacity (v/v, %)	Foam stability (v/v, %)	thermal properties	References
MBPI	–	3.33 ± 0.57 g g^−1^	3.00 ± 0.00 g g^−1^	63.18 ± 0.38^a^	62.75 ± 0.43^a^	89.66 ± 0.57	80.83 ± 1.04^c^	157.90 ± 0.17°C^d^	(38)
				72.03 ± 0.53^b^	66.50 ± 1.37^b^				
MBPI	–	2.62 ml g^−1^	10.5	–	–	26	76.9	–	(35)
MBPI	61.6 ± 1.5/65.6 ± 2.1^e^	–	–	–	–	–	–	100.8 ± 1.1°C^d^	(32)
MBPI	70.6	–	–	–	–	–	–	–	(10)
MBPI	72 ± 4.44	163 ± 10.05%	113 ± 6.84%	41.10 ± 1.87	21.00 ± 1.29	110 ± 6.78	58 ± 3.58	–	(39)
Globulins	92–99	–	–	90–120	–	–	–	80.8–83.0°C^d^	(29)
8S globulin	51–75	1.37–2.14 g g^−1^	1.90–3.84 g g^−1^	1.54–5.55	45–78	47–106	39–97	–	(33)

MBPI, mung bean protein isolates.

a In distilled water

b in 3% NaCl

c after 15 min

d denaturation peak temperature

e untreated/heated.

### 2. WAC and OAC

WAC or OAC is defined as the absorbed amount of water or fat per gram of protein, as protein has both hydrophilic and hydrophobic properties to interact with water and oil in foods. WAC is a useful indication to predict moisture loss if protein isolates can be incorporated into various food products. And OAC can reflect the hydrophobic capacity of protein. A strong negative correlation was found between WAC and OAC (33).

Brishti et al. (38) determined that the WAC and OAC of MBPI were 3.33 g g^−1^ and 3.00 g g^−1^ proteins, respectively. The results imply that MBPI could contribute to the improvement of textural and sensory qualities during processing of fabricated foods because of the proteins capability of retaining water and reducing interfacial tension in an emulsion system. However, Du et al. (35) measured that the highest WAC was 2.62 mL g^−1^, and the OAC was from 9.5 to 10.5 g g^−1^ except at the protein concentration of 1.5%. Butt and Batool (39) reported that WAC and OAC of MBPI were 163 and 113%, respectively (Table 3). In these three reports, similar preparation procedures of MBPI were applied as base extraction and acid precipitation. Liu et al. (33) determined that average WAC and OAC of mung bean 8S globulin were 1.92 g g^−1^ and 3.07 mL g^−1^, respectively. The varied values of WAC might be due to the protein structure and amount of polar amino acids, whereas the OAC difference might be due to the difference in nonpolar side chains binding the oil.

### 3. FC and FS

Foamability depends on air–liquid interface hydrophobicity, flexibility of protein molecules, protein solubility and denaturability (40). FC describes the stabilising ability of proteins by the amount of interfacial area per unit weight or concentration, and is related to molecular flexibility, charge density and hydrophobicity. And FS is the stabilising ability of proteins to the foam against gravitational and mechanical stresses as the effectiveness of whipping agents relies on their capability to preserve the whip as long as possible, which is affected by rheological properties of protein films, film elasticity and the magnitude of disjoining pressure between the protein layers (40).

Brishti et al. (38) reported that FC and FS of MBPI were 89.66 and 80.83% after 15 min of standing time, respectively. Butt and Batool (39) reported that FC and FS of MBPI were 110 and 58%, respectively. Due to different blending methods and different concentrations of MBPI, Du et al. (35) reported MBPI with 26% FC and 76.9% FS after 10 min of standing time, respectively (Table 3). This lower FC value might be caused by the high levels of hydrophobic amino acids after homogenisation at 10,000 rpm for 1 min. On the contrary, high FS could be due to the formation of a cohesive continuous network with high elasticity by MBPI, which shows optimum intermolecular interactions and creates stable foams at the air–liquid interface. Liu et al. (33) found significant positive correlation between FC and FS of mung bean 8S globulin with average 69.63% FC and 61.61% FS, respectively.

### 4. EA and ES

Emulsifying properties of proteins are also affected by the adsorption ratio of protein at the oil–water interface, the adsorbed amount of protein, interfacial rearrangement of conformation, the reduction degree in interfacial tension and formation of cohesive film (40). In a stabilised solution, EA represents the maximum interfacial area per unit weight of protein, and ES is the measure of the steadiness of emulsion formed by protein.

In 3% NaCl and in distilled water, EA and ES of MBPI were found to be 72.03 and 63.18, and 66.50 and 62.75%, respectively (38) (Table 3). Butt and Batool (39) reported that EA and ES of MBPI was 41.10 and 21%, respectively (Table 3). Liu et al. (33) determined that average EA index and ES of mung bean 8S globulin from different mung bean cultivars were 3.46 m^2^ g^−1^ and 63.15%, respectively. In chopped and fabricated meat-based products, EA and ES are critical factors. Since EA and ES of MBPI had been rather high comparing to other legumes (38), MBPI can be applied in both formation and stabilisation of fluid emulsion during the production of heat-treated textured vegetable proteins.

### 5. Thermal properties

The thermal properties of proteins, for example, denaturation of proteins, are often determined as an endothermic peak on the thermogram by DSC (41). Brishti et al. (38) reported that the denaturation temperature of MBPI was at 157.90°C, which is a transition temperature accompanied by rupture of intramolecular bonds when MBPI are heated from native to denatured state. Tang et al. (29) determined that the denaturation temperature of mung bean globulins was from 80.8 to 83.0°C (Table 3). Tang et al. (40) found that disulphide bonds within the protein molecule contribute to the thermal stability of protein. Kudre et al. (10) stated that the high thermal stability attribute could be due to the disulphide bonds, whereas the presence of salt bridges in the hydrophobic clefts of protein structure makes it more thermostable. In the process of optimisation of temperature, such as extrusion and heat treatment, the analysis of thermal properties of MBPI serves as an important tool.

## Extraction of mung bean proteins and peptides

Extraction of mung bean proteins has been widely studied. As a key step in mung bean protein research, many methods of protein extraction have been established. These methods are classified into two types, salt extraction methods and methods consisting of base extraction and acid precipitation. In order to isolate specific mung bean proteins, additional steps have been added to separation procedures, for example, heat treatment and Sephadex G-50 separation were used as additional steps for trypsin inhibitor isolation (24).

### 1. Salt extraction method

Johns et al. (42) reported an extraction method using a saturated ammonium sulphate solution. Mung bean proteins have also been extracted in 5% NaCl solutions, which was the optimum NaCl concentration to achieve a maximum total protein dissolution of 87.5%. Globulins have also been precipitated using saturated ammonium sulphate solution with concentrations of 20 and 65% for precipitating α-globulin and β-globulin, respectively, after which albumin was recovered from the dialysed supernatant liquid and isolated by coagulation. The yields of α-globulin and β-globulin were 0.35 and 5.75% (w/w), respectively, based on the dry weight of mung bean flour extracted, whereas the albumin yield ranged from 0.02 to 0.05% (w/w) of dry flour weight.

Using a different strategy, Rahma et al. (43) used a salt extraction method involving micellisation of mung bean proteins from a 0.5 mol L^−1^ sodium chloride water solution. The procedure consisted of extraction, centrifugation, filtration and micellisation steps. The micellised protein was separated by centrifugation, washed with water and redissolved at pH 7 by addition of sodium hydroxide. Basic 7S globulin was subsequently found to be easily extracted with 0.15 mol L^−1^ NaCl, whereas 11S globulin was extracted using 0.35 mol L^−1^ NaCl.

### 2. The method of base extraction and acid precipitation

Rahma et al. (43) also established a method of base extraction and acid precipitation, and compared it to the salt extraction method outlined above. Briefly, the procedure included alkaline water extraction at pH 8 (using a flour to water ratio of 1:20 w/v) followed by isoelectric precipitation at pH 4.5, washing, re-dissolution at pH 7 using 0.1 mol L^−1^ sodium hydroxide and centrifugation. Ultimately, 7S globulin, a predominant storage protein, was isolated using this method and 11S globulin was also isolated and determined to be a disulphide-linked polypeptide chain.

Comparative analysis suggested that the salt extraction method was much better for enrichment of the 7S globulin. Meanwhile, Thompson et al. (12) reported another method of base extraction and acid precipitation for the preparation of MBPIs. Numerous parameters, including pH, temperature, extraction time and ratio of mung bean flour to solvent, were optimised to increase the yield of protein extracted from mung bean flour. The highest yield of mung bean protein was achieved with the following optimised parameters: extraction at pH 9 at 25°C for 20 min with a 1:15 ratio of mung bean flour to solvent, followed by precipitation at pH 4. Subsequently, Kudre et al. (10) and El-Adawy (44) reported similar methods for MBPI whereby mung bean seed flour was extracted with NaOH (pH 12) or 0.1 mol L^−1^ NaOH (pH 9), respectively. Next, both extractions were precipitated with HCl (pH 4.5), followed by centrifugation and washing. Kudre et al. obtained approximately 87.8% of MBPI from dry mung bean seeds, whereas El-Adawy reported an average yield of 13 g protein/100 g mung bean flour.

In addition, other methods incorporating additional steps have been used to isolate specific bioactive proteins, such as trypsin inhibitor protein (24) and nsLTP (25). Klomklao et al. (24) found that the highest trypsin inhibitor activity of 822.63 unit g^−1^ seed and specific trypsin inhibitor activity of 31.95 unit mg^−1^ protein were obtained using distilled water extraction. Moreover, certain salt and alkaline conditions were observed to increase protein solubility, leading to a reduction in specific trypsin inhibitor activity [as observed in other studies of other legumes (45–47)]. Subsequently, a final specific inhibitory activity of 406 unit mg^−1^ protein was achieved after heat treatment, ammonium sulphate precipitation (30–65%) and Sephadex G-50 isolation. Ultimately, trypsin inhibitor activity was higher by about 13-fold compared to crude extract, with a yield of purified trypsin inhibitor from the extract of 30.25%. Meanwhile, Wang et al. (25) extracted anti-fungal nsLTP from ammonium sulphate precipitates coupled with purification using both CM-Sephadex C-50 and POROS-HS chromatography to isolate purified nsLTP, yielding 13 mg nsLTP from 100 g mung bean seeds.

## Bioactivities of mung bean proteins and peptides

Many different kinds of bioactive proteins and peptides have been reported from mung bean seeds. These proteins and peptides exhibit bioactivities, which may be beneficial to human beings and animals, including ACE inhibitors, trypsin inhibitor and anti-fungal agents.

### 1. ACE inhibitory activity

Several diverse biological pathways are known to regulate blood pressure in living organisms. One pathway, the renin angiotensin system, has been demonstrated to be acted upon by hypotensive peptides (31). Within the renin–angiotensin system, conversion of angiotensinogen to the pre-hypertensive hormone angiotensin I (DRVYIHPFHL) occurs through the action of renin secreted by the kidneys. Angiotensin I is further converted by ACE to angiotensin II (DRVYIHPF), which is the active form of the hormone, by ACE. Angiotensin II raises blood pressure by acting directly on blood vessels, sympathetic nerves and adrenal glands (48). Inhibition of ACE by ACE inhibitors is a strategy used to control hypertension.

ACE inhibitory activity is one of the main bioactivities reported for plant food-derived peptides (49). Aluko (50) reported that hydrolysed proteins from three legume sources, including mung bean, could provide ACE inhibitory activity. Indeed, food-derived ACE inhibitory peptides may be an alternative to synthetic drugs, since peptides are thought to cause fewer side effects (51). Consequently, Li et al. (52) have demonstrated that MBPI hydrolysed by Alcalase™ showed ACE inhibitory activity at a half maximal inhibitory concentration (IC_50_) of 0.64 mg protein mL^−1^. Using this method, the highest ACE inhibitory activity (53) was observed for a hydrolysate generated by Alcalase™ after 2 h of hydrolysis.

Meanwhile, another study demonstrated that a significant decrease in systolic blood pressure was observed in spontaneously hypertensive rats after ingestion of hydrolysed mung bean peptides. More recently, Li et al. (54) isolated three antihypertensive peptides from an Alcalase™-hydrolysated mung bean preparation using Sephadex G-15 and reverse-phase high-performance liquid chromatography (RP-HPLC) purification steps. The three peptides were identified by amino acid composition analysis and matrix-assisted-laser desorption/ionisation time-of-flight tandem mass spectrometry (MALDI-TOF MS/MS), as KDYRL, VTPALR and KLPAGTLF, with IC_50_ values of 26.5, 82.4 and 13.4 μM, respectively. Concurrently, using a different strategy, *Lactobacillus plantarum* B1-6-fermented mung bean milk was shown to produce significantly higher ACE inhibitory activity (67.5%) at the end of fermentation. Production of inhibitory peptides coincided with the disappearance of larger/more hydrophobic peptides with the appearance of increasing amounts of smaller/more hydrophilic peptides using RP-HPLC (55).

Using a different strategy, Mamilla et al. (56) compared mung bean grain and germinated seeds for ACE inhibitors using an *in vitro* ACE inhibition assay. Protein hydrolysates of germinated mung bean seeds showed greater than 82% ACE inhibition, reaching an IC_50_ value of 0.025 mg mL^−1^. However, the activities of ACE inhibitory peptides *in vitro* based on chemical tests are not always mirrored by their hypotensive effect *in vivo* on animal studies (57). Therefore, most of the presented studies should be treated with great caution due to the general poor correlation between *in vitro* biochemical assays on ACE and physiological responses *in vivo*. After all, many of the peptides, which show good activity *in vitro*, are degraded in the GI system, hence, explaining the unreliability of the assay results.

### 2. Trypsin inhibitory activity

Proteinase inhibitors, especially food-additive grade inhibitors, are in demand for protecting myofibrillar proteins from proteolysis by endogenous proteinases. Such inhibitors from legume sources, which generally inhibit trypsin, are safe, effective, thermally stable and inexpensive (10). For example, due to their inhibitory ability toward proteinases, trypsin inhibitors from legume seeds have been used for prevention of softening of mince or surimi gel mediated by heat-activated proteinases that are abundant in fish muscle or surimi (58). Subsequently, Sun et al. (59) reported that mung bean trypsin inhibitor additives were effective in preventing softening of surimi gel from marine fish blue scad. The fact that trypsin inhibitors from mung bean seeds are safe and effective in inhibiting trypsin activity suggests that their use as ingredients in drug formulations may prevent trypsin hydrolysis during drug administration.

Studies of purified mung bean trypsin inhibitor are beginning to shed light on this protein’s specific functions. In one study, Chrispeels et al. (60) reported trypsin inhibitory activity from the mung bean seed extracts prepared by a method of base extraction and acid precipitation followed by trypsin-sepharose affinity chromatography. They found that the purified trypsin inhibitor was not a double-headed inhibitor containing inhibitory sites for both trypsin and chymotrypsin, as observed in soybean trypsin inhibitor (61). Moreover, an aliquot (2.5 μl) of the purified trypsin inhibitor solution corresponding to one unit of trypsin activity did not inhibit vicilin peptidohydrolase, the major endopeptidase in the cotyledons of mung bean seedlings. In another study, Lorensen et al. (62) reported that six species of trypsin inhibitors, one major (F) and five minor inhibitor species (A–E), were observed in mung bean seeds, with overall trypsin inhibitory activity reported to be equivalent to 1.8 U g^−1^ of dry seed weight. More recently, Wilson et al. (63) determined the sequences of trypsin inhibitors C, E, F using a combination of automatic solid-phase and manual sequencing techniques. Analysis of trypsin inhibitor F showed that it contains 80 amino acid residues and exhibits a high degree of identity with the other sequenced members of the Bowman-Birk family of protease inhibitors. Trypsin inhibitors E, E’ and C are derived from inhibitor F by limited specific proteolysis. Notably, the majority cleavage sites noted in the F–E–C–E’ inhibitors were found to occur at peptide bonds involving aspartyl residues. Currently, two sequences of trypsin inhibitors are reported in the UniProt Database (http://www.uniprot.org/), that is, Bowman-Birk trypsin inhibitor (UniProt ID: IBB_VIGRR) and trypsin inhibitor (UniProt ID: Q1WA44_VIGRA). High conservation of these sequences related to other trypsin inhibitors has been reported (Table 4).

**Table 4:** The sequences of the mung bean trypsin inhibitors reported in UniProt database

UniProt ID	UniProt sequence
IBB_VIGRR	SHDEPSESSE PCCDSCDCTK SIPPECHCAN IRLNSCHSAC KSCICTRSMP GKCRCLDTDD FCYKPCESMD KD
Q1WA44_VIGRA	MMVLKVCVLV VFLVGVTTAG MDLNQLRSSH HHDSSDEPSE SSEPCCDSCR CTKSIPPQCH CADIRLNSCH SACKSCMCTR SMPGKCRCLD TDDFCYKPCE SMDKDDD

### 3. Anti-fungal and/or antibacterial activities

The anti-fungal and antibacterial protein, nsLTP (a basic, 9.03 kDa protein), which displays anti-pathogenic activity, has been isolated from the mung bean (*Vigna radiata*) seeds (25). The nsLTP protein is able to bind and transfer a variety of very diverse lipids between membranes *in vitro* (48). The N-terminal sequence of nsLTP was determined to be MTCGQVQGNL AQCIGFLEKG G. It exerts anti-fungal action toward *Fusarium solani*, *Fusarium oxysporum*, *Pythium aphanidermatum* and *Sclerotium rolfsii* and antibacterial action against *Staphylococcus aureus*, but not *Salmonella typhimurium*. The lipid-binding ability of the protein is very similar to that of previously described lipid transfer proteins extracted from wheat and maize seeds, indicating that it possesses lipid transfer activity (64).

Ye et al. (65) isolated an anti-fungal protein, mungin, from mung bean (*Vigna radiata*) seeds. Interestingly, mungin, an 18 kDa protein, possesses a novel N-terminal sequence homologous to cyclophilins. The N-terminal sequence of mungin is PNPKVFFDMT IGGQPAGKIV FELFADTTPR TAENFRALTT GEKGVSRGRK PLHYHGSIFH R. Mungin was shown to have anti-fungal activity against *Rhizoctonia solani, Coprinus comatus* and *Botrytis cinerea* and to a lesser extent on *Mycosphaerella arachidicola* and *Fusarium oxysporum*. Mungin also displayed inhibitory activity against α- and β-glucosidases but not against HIV-1 reverse transcriptase and β-glucuronidase. It is noteworthy that mungin, as a cyclophilin-like anti-fungal protein, also exhibited anti-mitogenic activity (66).

## Conclusion

MBPI have been reported to possess a nutritional-balanced amino acid composition using recommended FAO/WHO guidelines, with the exception of a deficiency in except sulphur-containing amino acids. However, protein engineering technology has been applied to introduce additional methionine and cysteine residues into mung bean 8S globulin to boost the methionine percentage from 41 to 145%. In a similar way, a free sulfhydryl group (cysteine residue) was introduced and the presence of a new disulphide bond was confirmed in cysteine-modified globulin.

The functional properties of MBPI, that is, protein solubility, WAC, OAC, FC and FS, EA and ES, and thermal properties, are useful properties for food-processing applications. Studies on MBPI functionality have been carried out for the development of food industry applications.

Extraction methods have allowed purification of several mung bean proteins and peptides from mung bean flour, paving the way for functional analyses. Extraction methods that alter the ionic environment, combined with alkaline extraction and acid precipitation, have been used most often. Notably, varies extraction ratios of various mung bean proteins can be obtained using various strategies. Globulins, the major proteins present in mung beans, account for about 85% of total protein. The subunit structure, N-terminal amino acid sequence, structure and homology amongst the three distinct isoforms of 8Sα globulin have been described in detail. However, the reports on mung bean albumins need to be clarified.

In addition, bioactive proteins and peptides hold special interest due to their potential health benefits in addition to their known nutritional functions. Specifically, ACE inhibitor and anti-fungal activities of mung bean protein hydrolysates and peptides have medical use, whereas trypsin inhibitors in mung bean protein fractions may serve as additive for preventing food proteolysis. Therefore, mung bean proteins and their hydrolysates hold great promise as sources of compounds with significant nutritional, functional and bioactive potential uses in foods, pharmaceuticals, other products and processes.
